# Carcinome tubulo-papillaire chez une jeune de 17 ans: à propos d’un cas et revue de la littérature

**DOI:** 10.11604/pamj.2017.26.73.11132

**Published:** 2017-02-20

**Authors:** Adil Kallat, Hicham Ouazize, Otheman Fahsi, Hani Abousaleh, Hachem El Sayegh, Ali Iken, Lounis Benslimane, Yassine Nouini

**Affiliations:** 1 Service d’Urologie A, Hôpital Ibn Sina, CHU Rabat, Maroc

**Keywords:** Carcinome tubulo-papillaire, rein, néphrectomie élargie, Tubulopapillary carcinoma, kidney, enlarged nephrectomy

## Abstract

Les tumeurs tubulo-papillaires représentent 10% des carcinomes à cellules rénales. Elles sont plus fréquentes chez l’homme que chez la femme (sex ratio: 8/1). L’âge moyen se situe dans la sixième décade. Sur le plan anatomopathologique, on distingue deux types: le 1 correspondant à une prolifération de cellules basophiles et le type 2 qui correspond à une prolifération de cellules éosinophiles. Nous rapportant le cas d’une patiente de 17 ans sans antécédents pathologiques qui s’est présentée aux urgences pour des douleurs de la fosse lombaire droite et du flanc droit avec hématurie évoluant depuis deux jours avant son admission, le tout évoluant dans un contexte d’amaigrissement et d’altération de l’état général. L’examen clinique à son admission retrouvait une patiente en mauvais état général, apyrétique avec une tension artérielle à 110mmhg/70mmhg et une fréquence cardiaque à 110 battements par minute. L’examen abdominal objectivait à l’inspection une voussure du flanc droit avec défense et empattement à la palpation. La numération formule sanguine retrouvait un taux d’hémoglobine à 6 g/dl ayant nécessité une transfusion. Le scanner abdominal a mis en évidence une volumineuse formation tissulaire polaire inférieure rénale droite de 10 cm/7.8 cm avec épanchement liquidien péri-rénal de 17 mm d’épaisseur. Deux jours après son admission l’évolution a été marquée par une déglobulisation avec accentuation des douleurs abdominales, on décide alors de faire une néphrectomie d’hémostase. On a réalisé une néphrectomie totale élargie droite par abord sous costal avec à l’examen anatomopathologique un carcinome tubulo-papillaire de type 2. Le scanner thoracique réalisé ultérieurement n’a pas objectivé de localisations secondaires.

## Abstract (en)

Tubulopapillary carcinomas account for 10% of renal cell carcinomas. They are more frequent in men than in women (sex ratio:8/1). The average period is the sixth decade. There are two anatomopathological types:the 1 corresponding to a proliferation of basophilic cells and type 2 corresponding to a proliferation of eosinophilic cells. We report the case of a 17-year old female patient with no particular past medical history presenting to the emergency department with pain in the right lumbar fossa and in the right flank associated with hematuria evolving for two days before admission, with loss of body weight and deterioration of the general status. Clinical examination on admission showed poor general condition. The patient was apyretic with blood pressure of 110 mmhg/70mmhg and a heart rate at 110 beats per minute. Abdominal examination based on patient’s inspection showed curvature of the right flank with tenderness, and guarding on palpation. Hemoglobin levels in the blood were 6 g/dl requiring transfusion. Abdominal scan showed voluminous right renal inferior polar tissue formation measuring 10 cm/7.8 cm with peri-renal effusion of 17 mm thickness. Two days after admission patient’s evolution was marked by deglobulisation associated with accentuation of the abdominal pains. The patient underwent hemostatic nephrectomy. Total enlarged right nephrectomy was performed via subcostal incisions. Histological examination revealed type 2 tubulopapillary carcinoma. Chest CT scan did not show secondary tumors.

## Introduction

Les tumeurs tubulo-papillaires représentent 10% des carcinomes à cellules rénales. Elles sont plus fréquentes chez l’homme que chez la femme (sexe ratio: 8 /1). L’âge moyen se situe dans la sixième décade. Elles regroupent l’adénome papillaire et le carcinome tubulo-papillaire. L’adénome papillaire se développerait à partir de reliquats métanéphriques et peut être considéré comme le précurseur du carcinome tubulo-papillaire. Les tumeurs tubulo-papillaires sont souvent multiples et parfois bilatérales. Elles représentent le type histologique le plus fréquemment retrouvé chez les hémodialysés ayant développé une dysplasie multikystique acquise.

## Patient et observation

**Figure 1:**
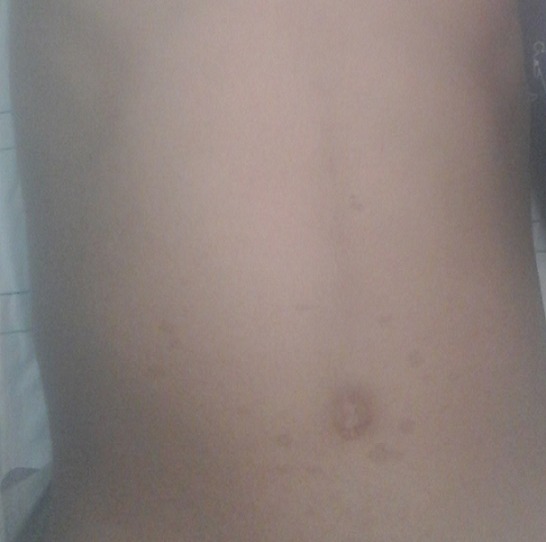
Voussure au niveau de l’hypochondre droit

**Figure 2:**
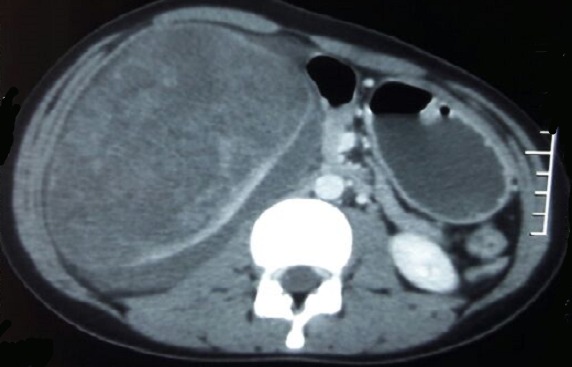
Scanner abdominal montrant une volumineuse formation tissulaire polaire inférieure rénale droite de 10 cm/7.8 cm avec épanchement liquidien péri-rénal de 17 mm d’épaisseur

**Figure 3:**
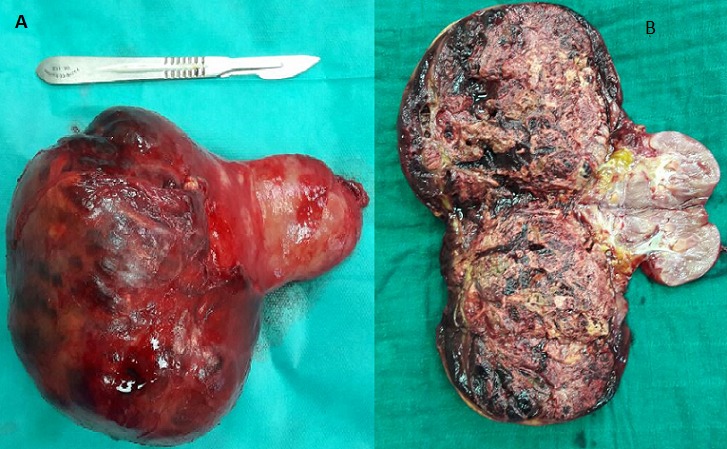
A) pièce opératoire de néphrectomie élargie intacte faisant 847g; B) pièce opératoire de néphrectomie élargie ouverte

**Figure 4:**
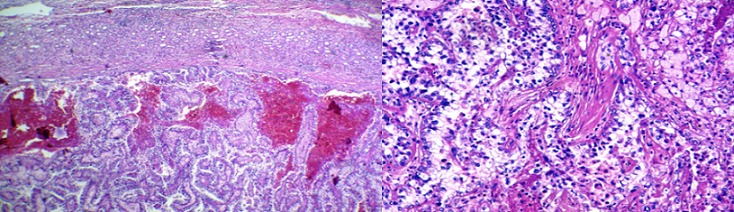
Carcinome tubulo-papillaire type 2: prolifération carcinomateuse d’architecture papillaire, les papilles sont bordées par plusieurs couches de cellules éosinophiles munis de noyaux discrètement irréguliers avec nucléole visible au fort grossissement

Nous présentant le cas d’une jeune patiente de 17 ans sans antécédents pathologiques qui s’est présentée aux urgences pour des douleurs de la fosse lombaire droite et du flanc droit avec hématurie évoluant depuis deux jours avant son admission, le tout évoluant dans un contexte d’amaigrissement et d’altération de l’état général. L’examen clinique à son admission retrouvait une patiente en mauvais état général, apyrétique avec une tension artérielle à 110mmhg/70mmhg et une fréquence cardiaque à 110 battements par minute. L’examen abdominal objectivait à l’inspection une voussure du flanc droit (Figure 1) avec défense et empattement à la palpation. La numération formule sanguine retrouvait un taux d’hémoglobine à 6 g/dl ayant nécessité une transfusion. Le scanner abdominal (Figure 2) a mis en évidence une volumineuse formation tissulaire polaire inférieure rénale droite de 10 cm/7.8 cm avec épanchement liquidien péri-rénal de 17 mm d’épaisseur. Deux jours après son admission l’évolution a été marquée par une déglobulisation avec accentuation des douleurs abdominales, on décide alors de faire une néphrectomie d’hémostase. On a réalisé une néphrectomie totale élargie droite (Figure 3) par abord sous costal avec à l’examen anatomopathologique un carcinome tubulo-papillaire de type 2 (Figure 4). Le scanner thoracique réalisé ultérieurement n’a pas objectivé de localisations secondaires.

## Discussion

L'individualisation des tumeurs tubulo-papillaires est relativement récente. Ce n'est qu'en 1976 que Mancilla-Jimenez réexamine 224 cas d'adénocarcinomes du rein, isolent 34 tumeurs à architecture tubulo-papillaire prédominante ou exclusive et établissent les premières particularités cliniques et radiologiques. Les tumeurs tubulo-papillaires représentent 10 à 15% des carcinomes à cellules rénales. Elles ont pour caractéristiques d'être souvent multiples (20 à 30%) et ont un meilleur pronostic que le cancer à cellules claires. Delahunt relance l'étude de ce type histologique en distinguant formellement en 1997 deux sous-types histologiques [1, 2]: le type 1 correspond aux tumeurs à cellules basophiles de petite taille avec cytoplasme réduit, petit noyau ovale, discret nucléole formant une seule assise de cellules le long de la membrane basale. Les macrophages spumeux y sont fréquents et les calcosphérites sont retrouvées dans 50%. Ce type est corrélé à des tumeurs de bas grade et stade de Robson et à une multifocalité importante. Le type 2 correspond aux tumeurs à cellules éosinophiles de plus grande taille avec cytoplasme abondant, noyau sphérique et large nucléole. Les macrophages spumeux sont rares et les calcosphérites retrouvées dans 10% des cas. Ce type est corrélé à des tumeurs de haut grade, apparaissant de plus grande taille, unifocales et corrélé à un moins bon pronostic. Dans une deuxième étude en 2001 [2], Delahunt compare 50 tumeurs tubulo-papillaires de type 1 à 16 tumeurs de type 2, et retrouve une cinétique de croissance plus importante pour les tumeurs tubulo-papillaires de type 2 et confirme leur corrélation à un grade plus élevé et à un moins bon pronostic. L’examen anatomopathologique de la pièce opératoire chez notre patiente a révélé, une prolifération carcinomateuse, d’architecture papillaire. Ces papilles sont bordées par plusieurs couches de cellules éosinophiles munis de noyaux discrètement irréguliers avec nucléole visible au fort grossissement (grade 2 de Furhman). Ce qui correspond à un carcinome tubulo-papillaire type 2. A l'examen tomodensitométrique, avant injection les tumeurs tubulo-papillaires ont un aspect homogène, à contours bien définis. L'interface avec le parenchyme environnant est nette. Classiquement la tumeur est iso ou hypodense par rapport au parenchyme adjacent. Ces lésions peuvent être le siège de calcifications dans un tiers des cas d'aspect punctiforme et de topographie centrale. Après injection du produit de contraste, le rehaussement est faible, uniforme et homogène [3]. Les tumeurs tubulo-papillaires apparaissent typiquement homogènes en isosignal en imagerie par résonance magnétique, mais peuvent se présenter en discret hypo ou hypersignal par rapport au cortex adjacent. Le rehaussement après injection du Gadolinium est faible et homogène. Il est significatif s'il dépasse 10 à 15 UH [4]. Nous pensons, comme plusieurs auteurs, que le traitement de référence reste la néphrectomie élargie du fait du risque de la récidive locale et de la multifocalité indétectable sur le bilan préopératoire [5].

## Conclusion

Les tumeurs tubulo-papillaires ont un meilleur pronostic que les carcinomes à cellules claires [6]. Mais selon des études récentes, il semble exister une hétérogénéité pronostique au sein de ces tumeurs; cette hétérogénéité pronostique serait liée à des paramètres cytogénétiques [7–10].
